# The quadratic relationship between difficulty of intelligence test items and their correlations with working memory

**DOI:** 10.3389/fpsyg.2015.01270

**Published:** 2015-08-24

**Authors:** Tomasz Smolen, Adam Chuderski

**Affiliations:** ^1^Neurocognitive Psychology Unit, Department of Psychology, Pedagogical University of KrakowKrakow, Poland; ^2^Cognitive Science Department, Institute of Philosophy, Jagiellonian UniversityKrakow, Poland

**Keywords:** fluid intelligence, working memory, Raven-test, correlation, floor/ceiling effects

## Abstract

Fluid intelligence (Gf) is a crucial cognitive ability that involves abstract reasoning in order to solve novel problems. Recent research demonstrated that Gf strongly depends on the individual effectiveness of working memory (WM). We investigated a popular claim that if the storage capacity underlay the WM–Gf correlation, then such a correlation should increase with an increasing number of items or rules (load) in a Gf-test. As often no such link is observed, on that basis the storage-capacity account is rejected, and alternative accounts of Gf (e.g., related to executive control or processing speed) are proposed. Using both analytical inference and numerical simulations, we demonstrated that the load-dependent change in correlation is primarily a function of the amount of floor/ceiling effect for particular items. Thus, the item-wise WM correlation of a Gf-test depends on its overall difficulty, and the difficulty distribution across its items. When the early test items yield huge ceiling, but the late items do not approach floor, that correlation will increase throughout the test. If the early items locate themselves between ceiling and floor, but the late items approach floor, the respective correlation will decrease. For a hallmark Gf-test, the Raven-test, whose items span from ceiling to floor, the quadratic relationship is expected, and it was shown empirically using a large sample and two types of WMC tasks. In consequence, no changes in correlation due to varying WM/Gf load, or lack of them, can yield an argument for or against any theory of WM/Gf. Moreover, as the mathematical properties of the correlation formula make it relatively immune to ceiling/floor effects for overall moderate correlations, only minor changes (if any) in the WM–Gf correlation should be expected for many psychological tests.

## 1. Introduction

Fluid intelligence (Gf) is an important cognitive ability that constitutes the main component of human general intellectual aptitude (Gustaffson, 1984). Gf consists of using reasoning (inductive, deductive, spatial, etc.) in order to solve novel abstract problems unsolvable by solely using existing knowledge. Fluid intelligence explains a large part of individual differences in the diverse types of human cognition and behavior. For instance, more intelligent people are better in knowledge acquisition, language comprehension, and spatial navigation, they achieve on average a higher socioeconomical status (including academic, professional, and financial one) than do less intelligent people, and they also better manage in daily life (e.g., less often meet with accidents, more effectively go through medical treatments, etc., Deary, 2012). A hallmark test of fluid intelligence is Raven's Advanced Progressive Matrices test, which requires discovering one or more abstract rules hidden within the geometrical pattern that is missing one fragment, and applying those rules in order to choose from several alternatives one correct solution that best matches the pattern.

An important theme in fluid intelligence research consists of identification of its underlying cognitive mechanisms. The last 20 years of research have produced convincing evidence that the strongest known predictor of Gf is the capacity (WMC) of working memory (WM)—a neurocognitive mechanism responsible for active maintenance and transformation of task-relevant information in the mind. Numerous studies have demonstrated that, when properly measured (see below), WMC can explain between half (Kane et al., 2005) and all variance in Gf (Oberauer et al., 2008; Chuderski, 2013). Unfortunately, this observation did not lead automatically to the identification of what makes both WM and Gf correlate strongly, because WM tasks are themselves quite complex; usually more than one WM process/resource is involved in performance in these tasks (Shipstead et al., 2014). Thus, one of the most exciting debates in Gf research concerns the identification of the mechanisms responsible for the strong association between WMC and Gf.

One influential theory assumes that shared variance in both WM tasks and Gf-tests depends on attention control exerted over cognitive processes that includes goal-driven directing attention and filtering out distraction (Kane and Engle, 2002; Shipstead et al., 2014). Evidence for this theory comes from significant correlations between Gf and the indices of executive control obtained from various tasks, for example involving memory updating (Burges et al., 2011), the inhibition of unwanted thoughts or prepotent responses (Dempster and Corkill, 1999; Unsworth et al., 2004), and dual-tasking (Ben-Shakhar and Sheffer, 2001). Moreover, research on high-WMC individuals (usually assessed with the complex span task that strongly correlates with Gf), compared to low-WMC people, demonstrated that the former were faster and more accurate on antisaccades (Unsworth et al., 2004), produced smaller error rates in incongruent trials using a high-congruent version of the Stroop test (Kane and Engle, 2003) and the flanker task (Heits and Engle, 2007), as well as more effectively suppressed distractors in a dichotic listening task (Conway et al., 2001). The attention-control theory of fluid reasoning holds that people with low attention control are poor reasoners because they find it difficult to maintain reasoning goals, and their cognitive processing is prone to frequent capture by irrelevant stimuli.

Alternatively, performance on simple *storage capacity* (short-term memory; STM) tasks that involve little attention control but require the active maintenance of a few items in parallel, was at least as good a predictor of Gf as performance on tasks requiring executive control, when rehearsal and chunking were blocked in the former tasks (e.g., Cowan et al., 2006; Colom et al., 2008; Chuderski et al., 2012). These results suggest that storage capacity may be the main determinant of fluid intelligence. One explanation (Carpenter et al., 1990) predicts that more capacious WM allows to keep the sub-products of reasoning (induced rules, elements of a solution, etc.) in the most active and accessible part of WM, called primary memory (Cowan et al., 2006). WM may also play an important role in fluid reasoning because it affects what relationships can be constructed among WM items (e.g., Hummel and Holyoak, 2003). Notably, Oberauer et al. (2008) proposed that *relational integration*—the construction of flexible, temporary bindings between a number of chunks held in WM in order to develop novel, more complex structures—is crucial to reasoning.

Although current theorizing tends to acknowledge that both executive control and storage capacity mechanisms in some way contribute to Gf (e.g., Cowan et al., 2006; Chuderski and Necka, 2012; Shipstead et al., 2014; Unsworth et al., 2014) the mutual relationships between these two mechanisms have not yet been understood satisfactorily (are they interacting or independent? does one underlie the other, or vice versa?), and it is still argued that either executive control (e.g., Burges et al., 2011; Shipstead et al., 2014) or storage capacity (Martínez et al., 2011; Chuderski et al., 2012) is a more fundamental factor for explaining fluid intelligence (whereas the other factor just explains some minor variance in Gf). One important set of arguments in favor of each theory came from the analysis of WM–Gf correlations in the function of an increasing difficulty of Gf-test items. Such studies empirically tested the hypothesis, originally put forward by the seminal capacity-based model of processing in the Raven-test (Carpenter et al., 1990), which assumed that more difficult items of the Gf-tests should involve more information being stored in WM, and thus such items should yield stronger correlations between Gf and WM than do easier items, when WM loads are unlikely to surpass the WMC of most participants. The logic of such tests was the following: if the storage-capacity account is right, then the positive correlation between the Raven item difficulty and WMC should be observed; otherwise, if no or even negative correlation is noted, then the storage capacity account should be rejected, and there is room for some alternative explanations of the neurocognitive basis of Gf (most possibly, the executive control or processing speed accounts).

Some researchers have indeed found evidence that Pearson's *r* increases for more difficult items (Little et al., 2014), and on this basis advocated the plausibility of storage-capacity account; whereas others found such correlations to be fairly constant (Salthouse, 1993, 2014; Unsworth and Engle, 2005; Salthouse and Pink, 2008; Wiley et al., 2011), and thus rejected this account, instead opting for the attention-control account. Moreover, a similar argument has been used outside the WM domain, for example in studies of relationships between intelligence and aging (Salthouse, 1993; Babcock, 2002) or learning (Carlstedt et al., 2000; Verguts and DeBoeck, 2002).

Our goal is to show that the above line of reasoning: if more difficult Gf items lead to stronger WM–Gf correlations, then storage capacity likely underlies Gf, if not, then some other mechanisms must underpin Gf, although intuitively attractive, is nevertheless fundamentally flawed. To outline our reasoning, the change of correlation for a particular item primarily depends on the amount of floor/ceiling effect for that item, and the maximal strength of correlation exists when no such effects are present. Thus, correlations drop for both very easy and very difficult items (i.e., a lot of floor/ceiling), and are the highest for items of average difficulty (little or no floor/ceiling). Moreover, the mathematical formula for the Pearson correlation is highly immune even to relatively large amounts of ceiling or floor effects if an overall WM–Gf correlation is moderate or weak, and thus only minor differences in correlation between easy/difficult and medium items can be expected for most of psychological tests. In consequence, no change in WM–Gf correlation with increasing Gf-test difficulty, or lack of it, can be used as an argument in favor of or against either the executive-control or storage-capacity accounts of WM/Gf. This matter is not only a statistical issue, but is a key methodological and theoretical problem, because the argument in question has been raised by numerous notable researchers in their theorizing about the WM and intelligence relationship (see below). Thus, it is crucial to systematically evaluate the validity of this argument.

The remaining text has been divided into four sections: First, we present the most notable examples in the literature of the arguments related to the increasing difficulty of Gf-tests. Second, using analytical inference we show the correlation between two variables to (slowly) decrease with increasing ceiling or floor effects. Third, with numerical simulations we demonstrate when the correlation formula is insensitive to such effects. Finally, we confirm our prediction empirically with the use of a large sample of participants, the Raven-test, and two methods of WMC measurement.

## 2. Examples of relevant studies

There are at least two cases in which correlation between two variables may potentially change between two conditions (e.g., easy and difficult). First, in Condition 1, the mechanism generating variance in variable *x* may also generate a substantial variance in variable *y*, but it may yield less or no such variance in Condition 2 (we will develop this argument formally in the subsequent section). Thus, in Condition 1, the variance of variable *y* consists of a relatively large amount of variance shared with variable *x*, and a relatively small amount of error variance (reflecting measurement errors and the effects of all other variables affecting variable *y* not accounted for). In Condition 2, however, the variance of variable *y* consists mainly of error variance, whereas the amount of variance shared with variable *x* is relatively small. As the correlation coefficient reflects the squared root of shared variance, such a coefficient will be substantially larger in Condition 1 than in Condition 2. For example, in our recent study (Chuderski and Necka, 2012), we found that scores on the well-known WM task, namely the *n*-back task, depend on either primary memory, when the WM load is small, or activated long-term memory (LTM), when the load is large (and the to-be-detected target falls out of the primary memory). Then, we found that the primary-memory condition substantially correlated with scores on intelligence tests (*r* ≈ 0.5), whereas in the LTM condition this correlation was close to zero. Thus, we concluded that primary memory underlies intelligence, whereas LTM is not an important mechanism for Gf. In general, this type of argument is quite obvious, and a substantial part of psychological knowledge has been inferred with such a logic.

However, the studies that raised the argument related to the increasing WM–Gf correlation with increasing difficulty of Gf-tests relies on quite a different logic. These studies assume that the same mechanism drives variable *y* in both conditions. However, it is expected that in an easier condition the ceiling effect will arise, and because this will yield less variation in data, it will also yield a lower correlation between variables, than will a more difficult condition in which ceiling effects will be reduced or absent (and the floor effect will still be low).

For example, investigating relations between intelligence, aging, and WM, Salthouse (1993, see also Salthouse and Pink, 2008; Salthouse, 2014) found WM–Gf correlations fairly stable for the Raven items that differed in difficulty:

Average solution accuracy varied considerably across the items examined, and it seems reasonable to hypothesize that at least some of the item variation might have been due to increased working memory demands. […] The configuration of results […] presents a challenge for interpretation. On the one hand, there is evidence of moderate to large relations between the measures of working memory and matrix reasoning performance, but on the other hand, the data indicate that these relations are no greater for difficult (low accuracy) than for easy (high accuracy) problems. A […] potential explanation is that much of the variation in item difficulty may be attributable to factors unrelated to working memory (pp. 181–182).

Similar arguments can be found in Unsworth and Engle (2005), who divided scores on the Raven-test items into four quartiles according to decreasing mean accuracy, and found constant correlations of the first three quartiles with a variant of complex span task:

[…] the correlation between solution accuracy and a measure of working memory capacity should increase as the number of rules, goals, and/or sub-results on a given problem increases (given that there is enough systematic variability present). That is, items with low memory loads will not exceed even the capacity of low WM span participants and thus most individuals should get these problems right and there should be little systematic variability present. However, as memory load increases so will item discriminability and thus the item-WM span correlations will increase (p. 70).

[…] the results suggest that, for the most part, the [WM–Gf] correlations are fairly constant and do not vary systematically with variations in memory load […] Taken together, the results of the present study strongly suggest that the number of goals or sub-results that can be held in memory does not account for the shared variance between working memory span measures and fluid intelligence. Thus, the results do not support the hypothesis […] that the link between individual differences in working memory capacity and intelligence is due to differences in the ability to hold a certain number of items in working memory (p. 78).

Furthermore, Wiley et al. (2011) used constant point-biserial correlations between progressively more difficult items of the Raven-test and a variant of the complex span to conclude:

Our results […] are consistent with previous findings in that neither the normative difficulty of RAPM items nor the number of rule tokens required for solution showed the positive relation with WMC that would be predicted by a rule/capacity explanation. […] these factors do not seem to be what drives the relation with WMC. […] Thus, differences in the quality of executive function, and not capacity *per se*, may be responsible for the relationship between WMC and RAPM (p. 261).

All these arguments against the storage-capacity account of Gf have often been referred to in the literature. To give only one example from Vigneau et al. (2006):

A recent study by Unsworth and Engle (2005) showed that the relation between working memory capacity and the Raven seems to be rather constant across levels of difficulty and memory load. This result is incompatible with the view that the number of rules or rule instances required to solve an item is central to the expression of individual differences on the Raven (p. 262).

Proponents of the storage-capacity account (Little et al., 2014) defended their approach by showing that the constant WM–Raven correlation in previous studies resulted from measurement errors and the generally low level of observed correlations between the Raven-test items/quartiles and WM scores. Obtaining pronounced such correlations, they showed that a relatively small but significant rise in the WM–Raven correlation can be observed when the Raven items become more difficult:

High overall accuracy lowers the item-wise correlation for the early items (i.e., the point-biserial correlation must be near zero if nearly everyone gets the item correct) resulting in an increasing slope across the entire test. For the later more difficult items, the participants who respond correctly have to come from the pool of participants who have higher Raven's scores and higher WMC. Consequently, with a high overall correlation between WMC and Raven's, the point-biserial correlation between WMC and the most difficult Raven's items that have the lowest accuracy, must be higher than the point-biserial correlation between WMC and the easiest Raven's items (p. 6).

When there is a moderate to strong overall correlation between WMC and performance on the Raven's-test of fluid abilities, then the role of WMC becomes increasingly more important as item difficulty increases. […] Our results are compatible with theoretical analyses of Raven's performance that appeal to working memory as a repository for rules and intermediate results (pp. 10–11).

From above citations we can clearly see that whether or not the increase in difficulty of Gf-test items led to an increase in their correlation has served as an important argument in the debate between attention-control and storage-capacity (and other) accounts. However, can the presence/lack of such an increase serve as an argument in favor/against the role of storage capacity in Gf? With a simple formal analysis, we aim to show that the answer to this question is definitely “no.”

## 3. Analytical inference of a relationship between test difficulty and its correlation with another test

### 3.1. General assumptions

In general, the issue of the analysis of correlations between different types of data is quite complex. However, we can make a few assumptions that effectively simplify the reasoning. Some of the assumptions have no effect on the generalizability of our reasoning. Other assumptions yield a small effect, but are reasonable on the grounds of empirical domain to which we refer.

1. All analyzed variables (independent variable, true test score and test outcome) display a mean equal to zero and a variance equal to one. This assumption is justified by the fact that the correlation estimator is scale independent and thus has no effect on reasoning.
2. All analyzed variables have the normal distribution.
3. A participant's ability does not depend on test difficulty.
4. Covariance of dependent and independent variables equals one. We assume so for the sake of simplicity of our analytical argument, but once our point is made, we show via numerical simulation that the same results still hold when this assumption is relaxed.
5. A test result is a linear function of ability and test difficulty unless a floor or ceiling effect arises (more details below).
6. The error term of the linear dependency between dependent and independent variables and the error term of the test result are independent.

**Figure 1:**
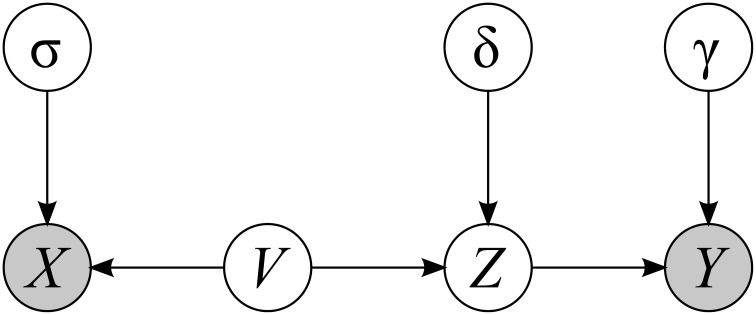
**Relation between random variables discussed**. Arrows indicate possible causal impact. White circles denote latent variables and gray circles denote observable variables. Lowercase Greek letters denote random noise.

We will refer to following random variables:

1. The true test score *Z* as it is defined on the ground of classical test theory (Guilford, 1954).
2. The independent variable *X*—the observable variable whose possible influence on dependent variable is examined. We suggest that the true independent variable is not observable, and what is observable is the sum of the true independent variable *V* and some random noise ζ (see Figure 1). However, since there path from *X* to *Z* is open, that is the causal impact flows from *X* to *Z*, (Pearl, 2009) we can treat variable *X* as possibly influencing dependent variable.
3. The observable test result *Y*—is a dependent variable.

Whenever we use uppercase Latin letter (e.g., *X*) we refer to random variable whereas the same lowercase letter (e.g. *x*) refers to a particular observation derived from that variable.

### 3.2. Impact of test difficulty

The observable test result *Y* is a sum of true score *Z* and error γ. We examine the relation between two variables: independent variable *X* and dependent test score *Z*. We assume there is a linear relation between *X* and *Z*:

Z=α+βX+δ,

where α and β are linear coefficients and δ is error. Variables *X* and *Y* are observable while true test score *Z* is latent.

Observed test results *Y* is sum of true test score *Z* and random noise γ:

Y=Z+γ.

Therefore, relation between *Y* and *X* is presented below:

Y=α+βX+δ+γ.

The linear coefficients α and β are scale dependent. As scale of the variables is of no interest here, we can assign to them values zero and one, respectively. Error terms δ and γ are independent and normally distributed and have a mean equal to zero. Thus, the sum ε = δ + γ of the error terms also is normally distributed and has a mean equal zero. If the variance of δ is equal σ^2^_δ_ and the variance of γ is equal σ^2^_γ_ then the variance of ε is equal σ^2^_δ_ + σ^2^_γ_. Thus, the relation between the observed test results and independent variable can be rewritten as follows:

(1) Y=X+ε.

The correlation between the set of observed results *Y* and the independent variable *X* influencing them is then negatively linearly related with error variance. It can be easily seen that when ε = 0 then *Y* = *X* and *r*_*X, Y*_ = 1, and when ε → ∞ then *r*_*X, Y*_ → 0.

**Figure 2:**
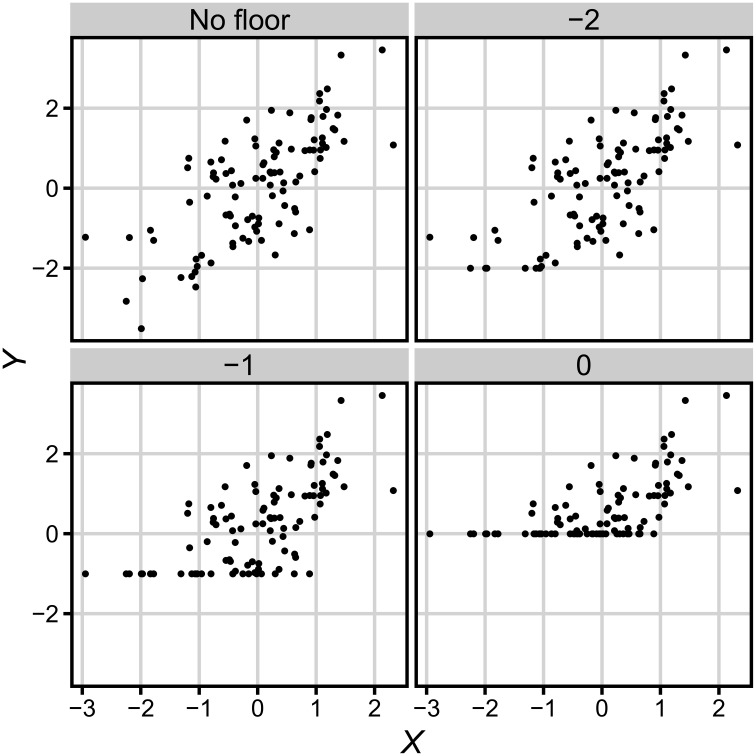
**Observed data *Y* for different values of floor *f***. See description in text.

We adopted following definition of test difficulty: when test difficulty increases, all results *Y* decreases proportionally to difficulty increase. We also assume that there exists floor value of test result *f* which is the minimal possible test score (see Figure 2). Thus, the extended relation between observed results and true scores takes form

(2) Y=max(X+ε,f).

That implies that if any observation *y* had value lower than *f*, then value *f* would be assigned to this observation. Depending on the amount of floor effect, a bigger or smaller part of data will equal *f*. The value of floor can be expressed in one of the two most convenient scales: as a standard score (in units of the distribution of *X*, where *f* is minimal possible value of scores, e.g., zero correct responses) or as a proportion of values on floor. As the former scale is more suitable for further inferences we will apply that scale, but one can easily transform *f* values to the other scale using the normal distribution function. Importantly, although our argument refers to floor effect and the increasing difficulty of a test, the very same argument symmetrically applies to ceiling effect and the decreasing difficulty of the test.

### 3.3. Analytical inference

Pearson product-moment correlation coefficient *r* between two random variables *X* and *Y* is a quotient of covariance of the variables and the product of their standard deviations:

$$r_{X,Y}=\frac{\text{cov}(X,Y)}{\sigma_{X}\sigma_{Y}},$$

or alternatively:

(3) $$r_{X,Y}=\frac{\sum_{i\;=\;1}^{n}\left(x_{i}-\bar{x}\right)(y_{i}-\bar{y})}{\sqrt{\sum_{i\;=\;1}^{n}{\left(x_{i}-\bar{x}\right)}^{2}}\sqrt{\sum_{i\;=\;1}^{n}{\left(y_{i}-\bar{y}\right)}^{2}}},$$

where *x*∈*X*, *y*∈*Y*, and *n* = |*X*| = |*Y*| is the number of observations. The covariance *cov*(*X, Y*) between two jointly distributed random variables *X* and *Y* is a measure of how strongly a value (relative to expected value) of one variable is linked to value (also relative) of the second variable, and it is defined as follows:

cov(X,Y)=E((x−E(x))(y−E(y))),

where **E**(*x*) means expected value of *x*. So covariance is the stronger the more observations vary from respective mean jointly on both scales and the weaker the more observations take high value on one scale and low value on the other one. As a change in test difficulty does not influence the participants' ability (Assumption 3), and the standard deviation of this ability equals one, the factor $1/\sqrt{\sum_{i\;=\;1}^{n}{\left(x_{i}-\bar{x}\right)}^{2}}$ in Formula (3) remains constant and equal one, so we can ignore it.

Correlation does not depend on data intercept; so as long as there is no floor, values *Y* can be increased or decreased without any change in correlation (in fact they can be manipulated in any linear way). Such a decrease in value *Y* would be what we have defined as the effect of an increase in difficulty. Nevertheless, the increase in difficulty would change the distribution of *Y* when this change causes some of the data points to reach floor. When some of the test results are on floor, whereas the independent variable remains unchanged, their correlation may alter in a non-linear way. So, let us examine what impact the relative change of *f* would have on Formula (3).

When floor is introduced, the distribution of test results *Y* becomes a mixture of two distributions: a truncated normal distribution (a normalized normal distribution with removed values below the threshold) with threshold *f*, and a degenerate (deterministic) distribution that includes only value *f*. From now on, the test difficulty will be defined as raising floor (and leaving *Y* unchanged) instead of decreasing *Y* (and leaving floor unchanged). These two approaches are fully equivalent, but the former is more convenient.

Proportion *p* of results belonging to the truncated normal distribution is a fraction of the normal distribution for values not lower than *f*:

$$p(f)=\int_{f}^{\infty }\phi (y)dy,$$

or alternatively:

$$p(f)=1-\frac{1+\text{erf}(\frac{f}{\sqrt{2}})}{2},$$

where ϕ(*x*) is the density of the standard normal distribution at *x* and *erf* is error function, which is a non-elementary function related to the cumulative normal distribution.

The mean value *y* of observed test result *Y* is an expected value of mixture of these two distributions with proportions *p*(*f*) and 1−*p*(*f*),

$$\bar{y}(f)=\int_{f}^{\infty }(\phi (y)ydy)+f(1-p(f)).$$

Whereas, the standard deviation σ_*Y*_ of observed test result *Y* equals

$$\sigma_{Y}(f)=\sqrt{\int_{f}^{\infty }(\phi (y){\left(y-\bar{y}(f)\right)}^{2}dy)+{\left(f-\bar{y}(f)\right)}^{2}(1-p(f))}.$$

Let us now consider covariance. As we assumed earlier, covariance of the variables analyzed equals one (as long as there is no floor effect). Therefore, the covariance cov(*X, Y, f*) of the joint distribution of *Y* and *X* for a given value of *f* is the sum of the weighted expected values of the products of differences between the values of the two distributions included in *Y* and their common mean *y*:

$$\begin{array}{l} \text{cov}(X,Y,f)=\int_{-\infty }^{f}\phi (y)y(f-\bar{y}(f))dy\\ \;+\int_{f}^{\infty }\phi (y)y(y-\bar{y}(f))dy. \end{array}$$

Note that because of the unit covariance *X* is entirely known given *Y* so there is no need to include *x* values in the formula [in fact the expression *cov*(*X, Y, f*) could be replaced by *cov*(*Y, f*)].

**Figure 3:**
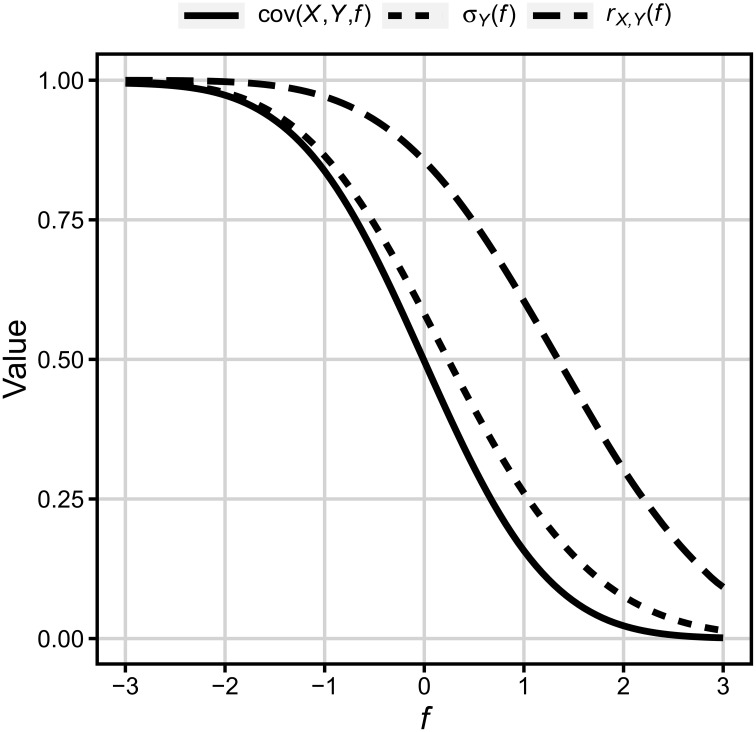
**Covariance, standard deviation, and correlation as functions of floor (in units of standard deviation of *X*, 0 = *x*)**. See description in text.

Figure 3 presents plot of three functions [cov(*X, Y, f*), σ_*Y*_(*f*), and *r*_*X, Y*_(*f*)] over floor. Values of floor are in standard deviation of *X*. Floor *f* = −3 means that all values of *Y* below −3 were replaced by the value of *f* analogously *f* = 0 means that half of the values of *Y* was replaced by 0.

In consequence, it can be easily seen that (a) the strength of correlation between two variables is a function of the amount of floor/ceiling effect for the dependent variable. However, (b) for any reasonable proportion of results on the floor/ceiling the correlation decreases relatively little in comparison to the case when no floor/ceiling effect is present. For example, for half results on floor/ceiling, which in psychology can be considered quite a strong floor/ceiling effect, *r*_*X, Y*_ drops from 1.0 to 0.86. The remaining part of the paper includes empirical tests of these two analytically derived predictions using both numerically generated and actually observed WMC and Gf variables.

## 4. Numerical simulations

Until now, we have considered only an idealized case of error term equal zero, that is, the case when *X* = *Y*. Let us focus on a more general case in which the amount of variance in error term influences correlation. Below, we analyze the change in correlation due to an increasing floor value in data generated by a numerical simulation.

Obviously, the correlation between *X* and *Y* is lower than unity when the error (ε) is larger than zero (see Formula 1). The larger the error, the less values *Y* become determined by values *X*, hence the covariance and correlation are lower. So, let us examine the influence of floor (*f*) on correlation (*r*_*X, Y*_) for different values of error.

**Figure 4:**
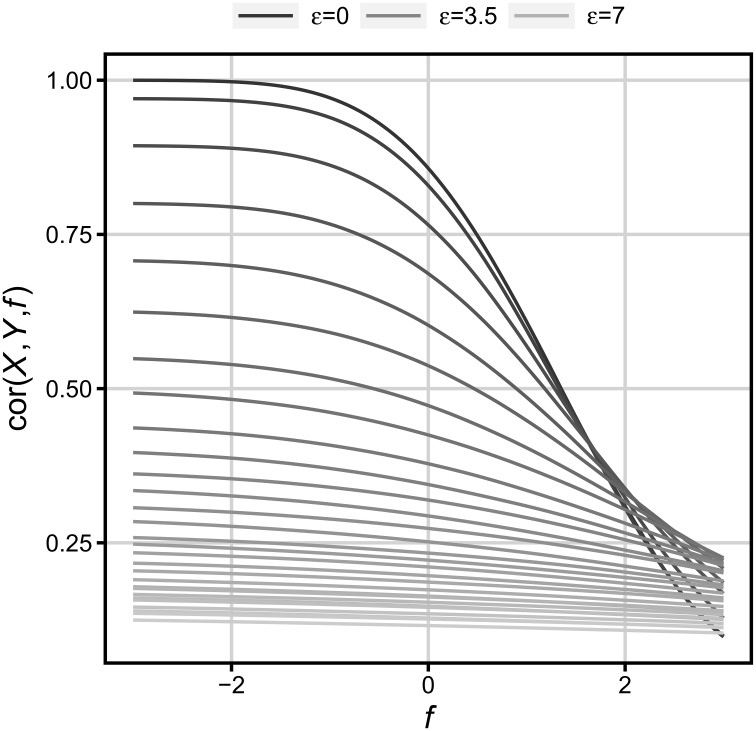
**Change in correlation over floor and error**. The error value differs from zero (the darkest highest line) to five standard deviations of *X* (the lightest lowest line).

In the present simulations, values *Y* were determined by a relationship depicted in Formula (1). Values *X* were drawn from the standard normal distribution. Values ε were drawn from normal distributions with the mean equal to zero, and variances systematically differing between 0 and 7. When floor is virtually absent (*f* = −3), these values yield correlations within the range between 1 and 0.18. Every sample comprised 100,000 observations. To determine the influence of floor on *r*, values of *Y* in every sample were transformed according to Formula (2) (one hundred values of floor were used). Figure 4 shows the changes in correlation over floor *f*, and error ε.

We can see that the lower the base correlation (i.e., when floor is absent), the less it decreases with an increasing floor level. For example, for the initial correlation equal to 0.5 that in psychology is a moderately strong relationship between variables, and for the floor level reaching 25 percent of cases (*f* = −0.67) that should also be considered a moderate floor effect, the correlation decreases from 0.5 to only 0.45. The same initial correlation (0.5) does not even reach 0.25 (less than a 50% decrease) until virtually all *Y* values reach floor (*f* = 2.61). Thus, it can be concluded that the correlation formula is highly immune to even large floor/ceiling effects, and moderate differences in floor/ceiling between task conditions need not result in any significant differences in correlation between these conditions, given that an overall correlation between WM- and Gf-tests is relatively low, or the sample is relatively small.

## 5. Empirical verification of the simulation results

The existing evidence on changes in the WM–Gf correlations when the floor or ceiling effects occur is mixed. Some studies did not find any such changes (Salthouse, 1993, 2014); some noted a slight decrease in correlations with increasing test difficulty (Unsworth and Engle, 2005; Wiley et al., 2011), whereas one study (Little et al., 2014) suggested a moderate increase. In this section, we aim to resolve this discrepancy by means of a theoretically-driven reanalysis of the results of our two recently published studies (Chuderski, 2013, 2015), administered to a large sample of participants (*N* = 939 in total). First, we analyzed data for *N* = 347 (from Chuderski, 2015) regarding the standard measure of WMC—the three variants of the complex span task (unfortunately, they were not used in the other study), as well as data from the Raven Advanced Progressive Matrices. Second, we used combined samples of *N* = 347 and *N* = 592 (the latter from Chuderski, 2013) and looked into data from the Raven as well as two alternative (strongly correlating with complex spans; see from Chuderski, 2015) measures of WMC—the short-term memory task and the relational integration task (that were used in both studies). Most participants were allowed 60 min. to complete the Raven (except for 288 people who were given 40 min). Due to relatively untimed testing, the participants had chance to attempt most items of the Raven. The overall correlation between the Raven and the mean from *z*-scores in three complex spans was *r* = 0.51 (*p* < 0.001;*N* = 347). Similar was the correlation between the Raven and the mean *z*-score of the short-term memory and relational integration tasks (*r* = 0.43, *p* < 0.001;*N* = 939). For participants data, procedure, descriptive statistics, reliabilities, and the correlations between tasks refer to Chuderski (2013, 2015).

### 5.1. Raven-test

The 36 items of Raven's Advanced Progressive Matrices (Raven et al., 1983, Section 4: Advanced Progressive Matrices) consist of a three-by-three matrix of figural patterns in which the bottom-right pattern is missing; subjects must choose a potential match for the missing pattern from eight response options (one option is correct). The task is to discover the rules governing the configuration of the patterns and apply them to select the single correct response option.

### 5.2. Complex span tasks

Adapted versions of three complex span tasks: the operation span, reading span, and symmetry span tasks were applied. Each task required participants to memorize a sequence of three to seven (i.e., set size) stimuli. Each stimulus, out of nine possible stimuli for that task, was presented for 1.2 s. Each stimulus was followed by a simple decision task, presented until a response was given, but for a maximum of 9 s. After two two-stimuli training trials, three trials for each set size (in increasing order) were presented in each complex span task. The operation span task analog required the memorization of letters, whilst deciding with a mouse button if an intermittent simple arithmetical equation (e.g., “2 × 3−1 = 5”) was correct. The modified reading span task consisted of memorizing digits, whilst checking if letter strings (e.g., “EWZTE,” “KTAN”) began and ended with the same letter. The spatial span task involved memorizing locations of a red square in the 3 × 3 matrix, whilst deciding which of two presented bars was larger. The response procedure in each task consisted of a presentation of as many 3 × 3 matrices as was a particular set size, in the center of the computer screen, from left to right. Each matrix contained the same set of all nine possible stimuli for a given task. A participant was required to point with the mouse at those stimuli that had been presented in a sequence, in the correct order. Only a choice that matched both the identity and ordinal position of a given stimulus was taken as the correct answer. The dependent variable for each complex span task was the proportion of correctly pointed stimuli to all stimuli in the task.

### 5.3. Short-term memory task

A variant of an array-comparison task was used that consisted of 90 trials. On each trial a virtual 4 × 4 array was filled with five to nine stimuli, picked from a set of ten Greek symbols (e.g., α, β, γ, and so on), then followed by a black square mask of the same size as the array, presented for 1.2 s, and then another array was shown. In a random 50% of trials, the second array was identical to the first; in the remaining trials the second array differed from the first by exactly one item in one position, which was always a new item (not a duplicate of an item from another position). The task was to press one of two response keys to indicate whether the highlighted item was the same or different in the two arrays. The task was self-paced.

### 5.4. Relation integration task

No-memory version of the alphanumeric monitoring task, originally devised by Oberauer et al. (2008), was used. The stimulus for each trial on the task consisted of a 3 × 3 array of syllables. Participants were asked to detect whether any of the rows or columns consisted of three syllables ending with the same letter. The array could either include one of the specified configurations; on these trials participants were required to press the space key to indicate that they had detected this configuration, or could not contain any of the specified configurations. Trials lasted 5.5 s and were followed by a 0.1 s blink separating subsequent arrays. There were 80 test trials.

### 5.5. Results and discussion

The mean scores for consecutive Raven items spanned from *M* = 0.92 to *M* = 0.09 (the floor defined by the theoretical random level was 0.125). All WM tasks yielded normal distribution and virtually no floor/ceiling effects.

First, for the sake of comparison with previous studies (Salthouse, 1993; Wiley et al., 2011; Little et al., 2014), we calculated the point-biserial correlations between each item of the Raven-test (ordered according to decreasing accuracy) and WMC (the mean of *z* scores in three complex tasks), for *N* = 347. As for the easiest Raven items a substantial ceiling effect existed, which disappeared for the medium items, whereas for the most difficult items a visible floor effect showed up (see Table 1), the goal of the analysis was to show that, consistently with our theoretical conclusions, the item-wise correlation between Raven and WMC would be increasing from the easy up to medium items, but it would start decreasing from the medium down to difficult items (as defined by error rate on particular items). However, in line with our above analyses, relatively slight (though significant) increases and decreases in correlation were expected.

**Table 1:** **Accuracy on the consecutive Raven-test items [with the 95% confidence intervals]**.

**Item**	**Accuracy**	**Item**	**Accuracy**	**Item**	**Accuracy**
1	0.87 [0.85, 0.89]	13	0.64 [0.60, 0.67]	25	0.50 [0.47, 0.54]
2	0.92 [0.90, 0.93]	14	0.80 [0.77, 0.83]	26	0.43 [0.40, 0.46]
3	0.90 [0.88, 0.92]	15	0.76 [0.73, 0.79]	27	0.39 [0.36, 0.43]
4	0.86 [0.84, 0.88]	16	0.78 [0.76, 0.81]	28	0.27 [0.25, 0.30]
5	0.86 [0.84, 0.88]	17	0.73 [0.70, 0.76]	29	0.24 [0.21, 0.27]
6	0.91 [0.89, 0.93]	18	0.67 [0.64, 0.70]	30	0.39 [0.36, 0.42]
7	0.89 [0.87, 0.91]	19	0.73 [0.70, 0.76]	31	0.34 [0.31, 0.37]
8	0.83 [0.80, 0.85]	20	0.69 [0.66, 0.72]	32	0.30 [0.27, 0.33]
9	0.91 [0.89, 0.93]	21	0.58 [0.55, 0.62]	33	0.37 [0.34, 0.40]
10	0.82 [0.79, 0.84]	22	0.55 [0.52, 0.58]	34	0.26 [0.23, 0.29]
11	0.91 [0.89, 0.93]	23	0.58 [0.55, 0.61]	35	0.35 [0.32, 0.38]
12	0.86 [0.83, 0.88]	24	0.41 [0.38, 0.44]	36	0.09 [0.07, 0.11]

**Figure 5:**
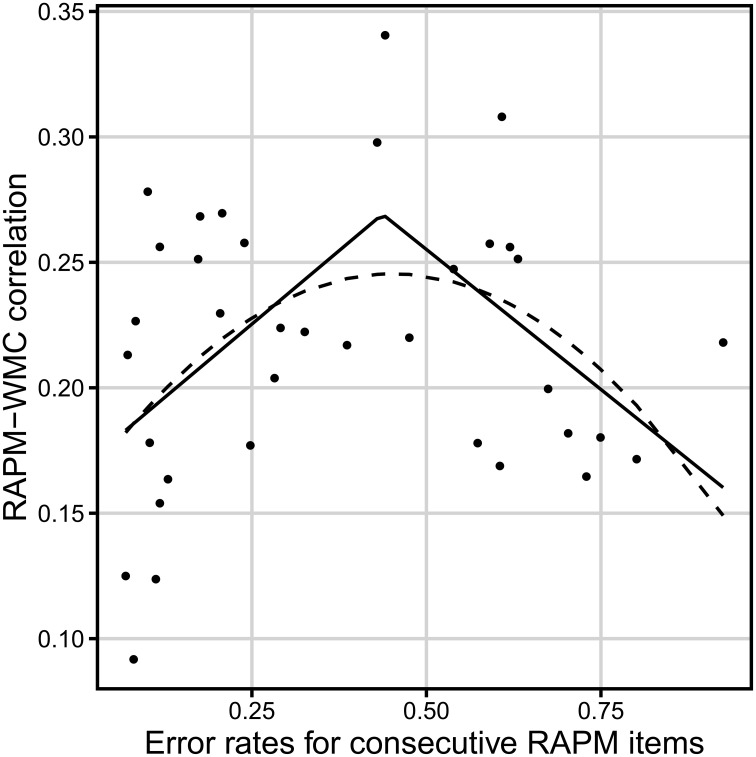
**Relation between the dificulty of a Raven-test item (error rate) and the correlation between accuracy on that item and WMC (measured with the complex span tasks)**. The solid line represents the segmented regression line. The dashed line reflects the quadratic regression line *N* = 347.

There was an insignificant linear dependency between error rate on a Raven item and its correlation with WMC score, *F*_(1, 34)_ = 0.4, *p* = 0.53 (see Figure 5). However, the segmented regression including the breakpoint (i.e., a different linear coefficients before and after the breakpoint) that optimized the fit revealed significant non-linear relation between difficulty of the Raven-test item and WMC score, adjusted *R*^2^ = 0.17 (see Table 2 for parameters, and Figure 5 for the fitted values). ANOVA test indicated that better fit of segmented regression over linear regression compensated larger complexity of the former, *F*_(2)_ = 4.73, *p* = 0.02. Correlation between error rate and the Gf–WMC correlation equaled *r* = 0.46 for error rate not higher than breakpoint of 0.44 (*n* = 21), while it equaled *r* = −0.52 for error rate above this breakpoint (*n* = 15). Also, a second degree polynomial model fitted better than a first degree polynomial model, *F*_(1)_ = 134.68, *p* < 0.001, (adjusted *R*^2^ = 0.14 and −0.02, respectively) and its predictions closely matched the predictions of the segmented regression (to the extent of difference in their shapes; see Figure 5).

**Table 2:** **The 95% confidence intervals for parameters of the regression analyses for the correlation of the Raven items with WMC (calculated from the three complex span tasks; *N* = 347) over difficulty of the items**.

**Regression**	**Parameter**	**Point estimation value**	**95% confidence interval**
Linear [*F*_(1, 34)_ = 0.4]	Intercept	0.21	[0.17, 0.24]
	Slope	0.02	[−0.05, 0.1]
Quadratic [*F*_(2, 33)_ = 3.78]	Intercept	0.16	[0.11, 0.21]
	Linear term coefficient	0.39	[0.1, 0.68]
	Quadratic term coefficient	−0.43	[−0.76, −0.1]
Segmented [*F*_(3, 32)_ = 9.95]	Intercept	0.17	N/A
	Breakpoint	0.44	[0.14, 0.61]
	Slope before breakpoint	0.23	[0.06, 1.79]
	Slope after breakpoint	−0.46	[−0.68, −0.04]

*Point estimation values are values of parameters fitted by regression which do not take in the account the error of estimation*.

**Figure 6:**
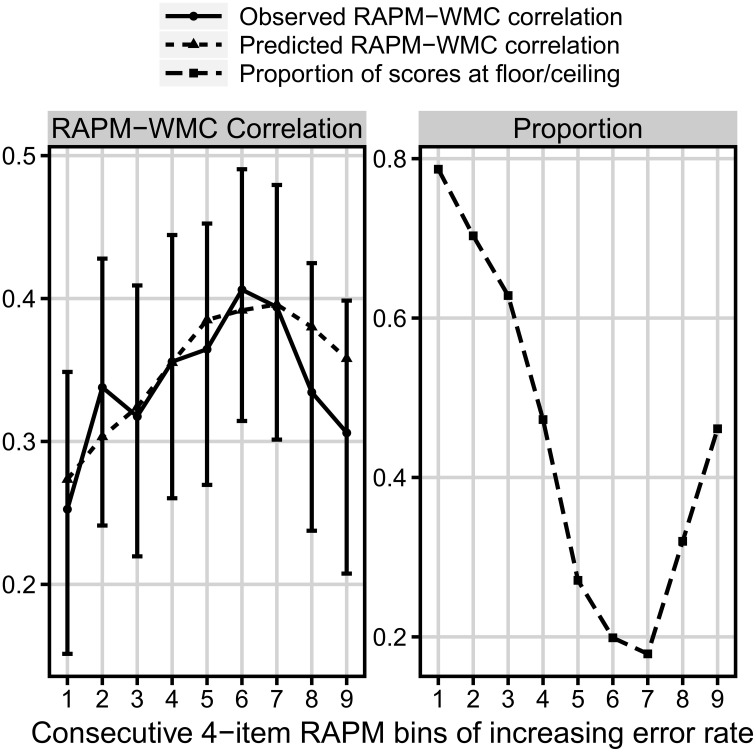
**Comparison of three measures as a function of the consecutive bins increasing in difficulty**. **Left:** the observed (solid line) and predicted (dotted line) bin correlation with WMC (measured with the complex span tasks). **Right:** the proportion of floor/ceiling effect (dashed line). *N* = 347

However, because each participant gave only one response to each Raven's item, we were not able to directly estimate the amount of floor/ceiling effects for single items. Instead, we computed ceiling/floor effect for nine bins of items (four items in each) constructed according to increasing difficulty of items. As can be seen in Figure 6, the observed correlations with WMC differed between the bins. The weakest correlation for the first bin was significantly weaker than the strongest correlation for the sixth bin, *z* = −1.86, *p* = 0.031, whereas the correlation for the latter bin was marginally stronger then the correlation for the last bin, *z* = 1.55, *p* = 0.06.

Furthermore, for each bin we used the proportion of scores with either four or zero correct responses as the proportion of either ceiling or floor effect for that bin. We used the proportion of the dominant effect in each bin as the measure of floor/ceiling effect level. Using our formal model, with such a measure we were able to predict specific values of correlation between accuracy in the consecutive Raven bins, and WMC (as a reminder: in the model the correlation with WMC in each bin depends on both the amount of floor/ceiling for that bin and the overall correlation between the variables in the no-effect case; see Figure 4). As we did not know the no-effect correlation, we fitted its value (it was the only parameter fitted). In result, the match between observed and predicted correlation was very good, *RMSD* = 0.028, *r* = 0.82, χ^2^_(8)_ = 3.31, *p* = 0.91 (see Figure 6).

**Figure 7:**
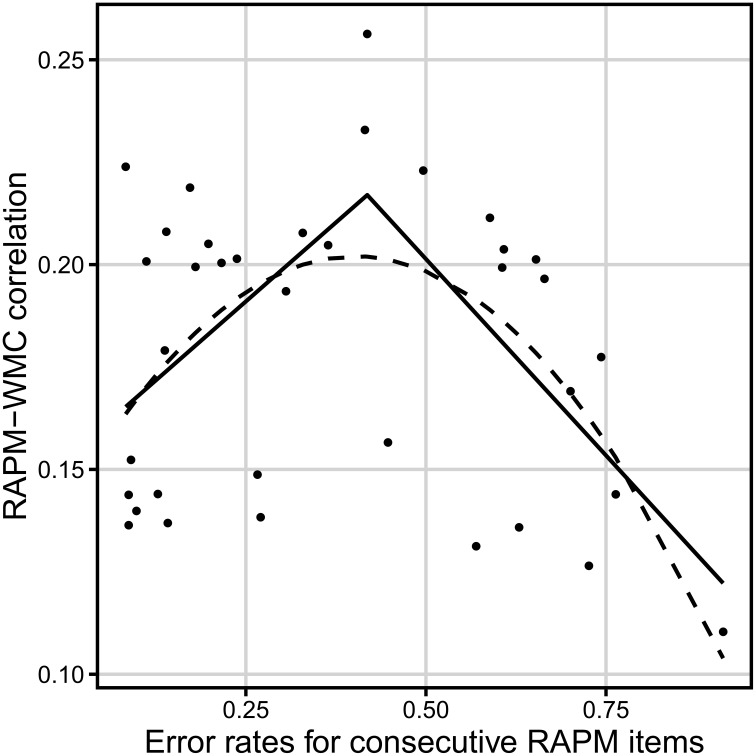
**Relation between the dificulty of a Raven-test item (error rate) and the correlation between accuracy on that item and WMC (measured by the short-term memory and relational integration task)**. The solid line represents the segmented regression line. The dashed line reflects the quadratic regression line *N* = 939.

We also repeated the above described single-item and bin analyses for the 939-people data, and the short-term memory and relation integration tasks (i.e., for the mean *z*-score on these two latter tasks). For the single items, exactly as in previous analysis, the segmented regression fitted the data much better than linear regression, *F*_(2)_ = 6.10, *p* = 0.006 (adjusted *R*^2^ = 0.22 and −0.013, respectively, see Table 3 for parameters, and Figure 7 for the fitted values). The breakpoint was detected at error rate equaling 0.42. Error rates before that breakpoint yielded a positive correlation with the Gf–WMC correlation coefficients (*r* = 0.53), whereas error rates after the breakpoint correlated negatively with the Gf–WMC correlation coefficients (*r* = −0.50). Also, similarly as for the complex tasks, a second degree polynomial model (adjusted *R*^2^ = 0.23) fitted better than a first degree polynomial model, *F*_(1)_ = 11.71, *p* = 0.002, and it gave the overall predictions quite compatible with the segmented regression output.

**Table 3:** **Confidence intervals for parameters of the regression analyses for the correlation of the Raven items with WMC (calculated from the short-term memory and relation integration tasks; *N* = 939) over difficulty of the items**.

**Regression**	**Parameter**	**Point estimation value**	**95% confidence interval**
Linear [*F*_(1, 34)_ = 0.55)	Intercept	0.19	[0.16, 0.21]
	Slope	−0.019	[−0.07, 0.032]
Quadratic [*F*_(2, 33)_ = 6.22]	Intercept	0.14	[0.11, 0.17]
	Linear term coefficient	0.31	[0.11, 0.5]
	Quadratic term coefficient	−0.38	[−0.6, −0.15]
Segmented [*F*_(3, 32)_ = 13.5]	Intercept	0.15	N/A
	Breakpoint	0.42	[0.17, 0.66]
	Slope before breakpoint	0.15	[0.0036, 0.65]
	Slope after breakpoint	−0.35	[−0.46, −0.065]

*Point estimation values are values of parameters fitted by regression which do not take in the account the error of estimation*.

**Figure 8:**
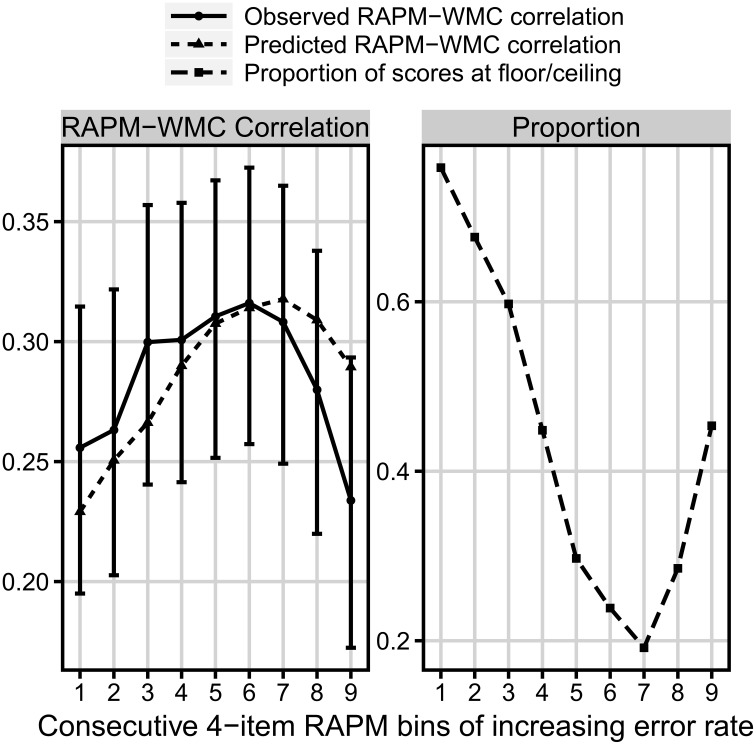
**Comparison of three measures as a function of the consecutive bins increasing in difficulty**. **Left:** the observed (solid line) and predicted (dotted line) bin correlation with WMC (measured by the short-term memory and relational integration task). **Right:** the proportion of floor/ceiling effect (dashed line) *N* = 939.

Finally, the analysis of the bins, constructed in the same way as for the *N* = 347 sample, but now for the *N* = 939 sample, yielded a non-linear relation between bin difficulty and its correlation with WMC, now with the latter variable measured in a different way. We predicted these data using our formal model, and received only a little bit worse match to the observed 939-people data than in the case of the *N* = 347 sample, *RMSD* = 0.026, *r* = 0.57, χ^2^_(8)_ = 6.87, *p* = 0.55 (see Figure 8), which might have resulted from the overall weaker correlation between Gf and WMC, when the latter was measured with the short-term memory and relational integration tasks.

Additionally, we were interested in testing validity of another claim supposed to reject the storage capacity theory, regarding differences between item-wise Raven and WMC correlations. Specifically, Wiley et al. (2011) reported much stronger item-wise correlations with the complex span task if the set of rules for a particular Raven item was used for the first time throughout the test (and thus possibly placed larger demands on executive control or rule abstraction), than when it was repeated from one of the previous items. Consequently, we used item coding from Wiley et al., resulting in 18 items coded as new-rule items, and 18 as old-rule ones (as we only used Raven's Set II). We used the 347-people dataset for the sake of compatibility of studies (i.e., both analyses pertained to Raven correlations with the complex span tasks). In our data, both correlations did not differ significantly, *z*_(34)_ = 1.41, *p* = 0.17 (mean *r* = 0.223 vs. *r* = 0.197, for the new- vs. old-rule items, respectively). Our data match the results of Little et al. (2014), who also failed to find any differences in WM predictive power between the new- and old-rule items. Moreover, in a recent design that prevented several confounds found in Wiley et al. and Little et al. studies (as well as in our study), Harrison et al. (2014) found a lower WMC–Gf correlation for the new-rule items than for old-rule items. Thus, Wiley et al.'s (2011) results seem to be an artifact, likely resulting from their use of only one variant of the complex span task (and, thus, a large amount of task-specific variance), as well as an overall low WMC–Gf correlation observed.

## 6. Conclusion

This paper aimed to investigate the commonly adopted assumption within the working memory and fluid intelligence research, which holds that if the storage capacity underlay the WM–Gf correlation, then such a correlation should increase with an increasing difficulty of a Gf-test, because more difficult test items are more sensitive to individual differences in WM. As often no such link is observed, on that basis the storage-capacity account is rejected, and other accounts (e.g., ones referring to executive control or processing speed) are favored. In contrast, when this link is found, it is used to support the storage-capacity account. Therefore, the above claim yields important implications for the current theorizing on the cognitive basis of intelligence. Our formal analysis demonstrated that the above assumption is incorrect, and reasoning that is derived from this assumption can speak neither for nor against the storage capacity account (nor any other account).

Specifically, using both analytical inference and numerical simulations, we have shown that the WMC–Gf correlation primarily depends on the amount of floor/ceiling effect for particular items/bins. Thus, whether the item-wise WMC correlation of a given Gf-test increases or decreases (or remains unchanged) with an increasing difficulty (error rate) of its items depends on the overall difficulty of the test, as well as the distribution of difficulty across its items. For easy progressive tests, in which the early items yield huge ceiling, but the late items do not approach floor, the item-wise WMC–Gf correlation will indeed increase throughout the test. In contrast, for difficult tests, in which the early items locate between ceiling and floor, but the late items approach floor, the respective correlation will decrease. For tests such as Raven, whose items span from substantial ceiling to substantial floor, the quadratic relationship will be observed. Fully confirming the predictions of our theoretical model, we demonstrated this latter relationship empirically, using large samples and two alternative methods of WMC measurement. Finally, for tests whose items vary in difficulty, but neither the easier items approach ceiling nor the harder ones approach floor, no significant differences in item-wise correlation with WMC will be observed. Thus, the investigated claim which holds that if the storage-capacity account was true, then the WM–Gf test correlations should increase simply as the function of items difficulty, does not seem justified. Vice versa, even observing exactly such an increase does not automatically support the storage-capacity account. Therefore, no changes in correlations due to differences in WM/Gf load (and resulting floor/ceiling effects), or lack of them, can be used as an argument for or against any theory of WM and/or Gf.

Another problem, cogently noted by a reviewer, pertaining to derivations of theoretical conclusions from the item-wise analyses of Gf-tests, is related to the fact that to date all such analyses relied on tests which include a very limited pool of items, presented in a fixed order. Thus, a given relationship between WMC and the order/difficulty of a Gf-test item may be obscured by unknown peculiarities concerning that item (e.g., an awesome rule), or the fact that the item was presented at a particular position within a sequence (e.g., late one, when a participant already became tired or subject to time pressure). Thus, a more correct way of testing the model presented here would be to use a test for which load-varied items are generated dynamically (i.e., their pool is large), and in the random order concerning their difficulty. However, as the use of such tests in literature is still rare (for some exceptions see Embertson, 1995; Primi, 2002; Arendasy et al., 2008), and the present study was primarily devoted to the critical evaluation of existing studies on the item-wise WMC/Gf analyses (that most widely used Raven APM), here we also focused on the Raven. However, the future testing of our model against data from a dynamically generated Gf-test (data still to be gathered) will definitely constitute a more powerful and general test of the model.

Although some scholars (e.g., Unsworth and Engle, 2005; Little et al., 2014) previously suggested that profound ceiling/floor effects may lead to decreased correlations, our paper is the first to elucidate the complex relationship between the amount of ceiling/floor for a Gf-test item, the Gf-test's overall strength of correlation with WM, and the resulting WM correlations for particular Gf-test items, as well as to test this relationship in a substantially large sample. Besides the significant contribution of our analysis to the evaluation of arguments for or against particular theories of WM/Gf, this work also sheds light on what is in fact measured by Gf-test items that differ in difficulty.

Although we focused on the relationship between WM and Gf, our line of reasoning pertaining to ceiling/floor effects and their impact on correlation can as well be generalized onto tests from other domains of psychology. Moreover, in line with Little et al. (2014) we conclude that the mathematical properties of the correlation formula make it relatively immune to the introduction of both floor and ceiling effects into the distribution of the dependent variable, when the overall strength of correlations is moderate, that is, lower than *r* = 0.7. As in psychology the usual correlation strengths fall between 0.2 and 0.6, it is likely that even if half the participants score near floor/ceiling level in a given part of psychological test, its correlation with any variable will decrease only by a few points (probably insignificantly), in comparison to the part in which floor/ceiling is absent.

## Author contributions

TS has carried proof of the main thesis, performed numerical simulation, and made statistical analysis of experimental data. AC has formulated the main thesis, framed and expressed the theoretical basis of the inference, performed experiments, and gathered data.

## Funding

The study was supported by National Science Centre of Poland (project 2013/11/B/HS6/01234).

### Conflict of interest statement

The authors declare that the research was conducted in the absence of any commercial or financial relationships that could be construed as a potential conflict of interest.
